# Infectivity of Wild-Bird Origin Influenza A Viruses in Minnesota Wetlands across Seasons

**DOI:** 10.3390/pathogens13050406

**Published:** 2024-05-14

**Authors:** Rebecca L. Poulson, Andrew B. Reeves, Christina A. Ahlstrom, Laura C. Scott, Laura E. Hubbard, Alinde Fojtik, Deborah L. Carter, David E. Stallknecht, Andrew M. Ramey

**Affiliations:** 1 Southeastern Cooperative Wildlife Disease Study, Department of Population Health, College of Veterinary Medicine, University of Georgia, Athens, GA 30602, USA; 2 U.S. Geological Survey, Alaska Science Center, 4210 University Drive, Anchorage, AK 99508, USA; 3 U.S. Geological Survey, National Wildlife Health Center, 6006 Schroeder Road, Madison, WI 53711, USA; 4 U.S. Geological Survey, Upper Midwest Water Science Center, 1 Gifford Pinchot Drive, Madison, WI 53726, USA

**Keywords:** avian influenza, environment, Minnesota, persistence, virus, water

## Abstract

The environmental tenacity of influenza A viruses (IAVs) in the environment likely plays a role in their transmission; IAVs are able to remain infectious in aquatic habitats and may have the capacity to seed outbreaks when susceptible wild bird hosts utilize these same environments months or even seasons later. Here, we aimed to assess the persistence of low-pathogenicity IAVs from naturally infected ducks in Northwestern Minnesota through a field experiment. Viral infectivity was measured using replicate samples maintained in distilled water in a laboratory setting as well as in filtered water from four natural water bodies maintained in steel perforated drums (hereafter, mesocosms) within the field from autumn 2020 to spring 2021. There was limited evidence for the extended persistence of IAVs held in mesocosms; from 65 initial IAV-positive samples, only six IAVs persisted to at least 202 days in the mesocosms compared to 17 viruses persisting at least this long when held under temperature-controlled laboratory settings in distilled water. When accounting for the initial titer of samples, viruses detected at a higher concentration at the initiation of the experiment persisted longer than those with a lower starting titer. A parallel experimental laboratory model was used to further explore the effects of water type on viral persistence, and the results supported the finding of reduced tenacity of IAVs held in mesocosms compared to distilled water. The results of this investigation provide evidence that many factors, including temperature and physicochemical properties, impact the duration of viral infectivity in natural settings, further extending our understanding of the potential and limitations of environmental-based methodologies to recover infectious IAVs.

## 1. Introduction

The environmental tenacity of avian influenza A viruses (IAVs) may play an important role in their maintenance and transmission. Wild birds, particularly those in orders of Anseriformes (ducks, geese, swans) and Charadriiformes (shorebirds, gulls), have long been considered the primary reservoirs for most of the IAV diversity that exists in nature. Wild birds have the capacity to shed high concentrations [1] of infectious IAVs into the environment through cloacal excretions, a mechanism posited to play an important role in the fecal–oral transmission of IAVs among wild birds. Intact viruses, if able to remain infectious for extended periods of time, may be able to reseed outbreaks over both short- and long-term scales. Incursions of highly pathogenic Gs/GD lineage H5 IAV into North America in late 2021–2022s [2,3,4,5] underscore the need to better understand the environmental tenacity of IAVs and the role persistence of IAVs in nature might play in the maintenance of viruses on the landscape and in waterborne transmission to susceptible hosts.

The persistence of IAVs in the natural environment and the potential role of such persistence in transmission dynamics are not fully understood. Reports of infectious IAVs isolated from the environment are rare and often in spatial and temporal association with large concentrations of waterfowl or gulls [6,7,8,9,10,11,12]. Furthermore, the methodology for detecting infectious IAV from environmental water samples remains inconsistent and unrefined. Seminal work utilizing distilled water laboratory models showed that low pathogenicity (LP) H10N7 IAV was able to remain infectious for prolonged periods of time at 4 °C and that IAV can persist for as long as 200 days at 17 °C [13]. Building upon this, later work in this same model system showed that while LP IAVs tend to persist best at slightly basic (7.4–8.2) and fresh to brackish (0–20,000 ppm) salinities, these relationships quickly become complicated when considering other physiochemical parameters at the same time [14]. Viral properties, such as strain and subtype variation, further complicate these interactions [14,15].

Translation and application of results derived from well-controlled lab-based studies to more complicated natural settings have proven difficult. Recent research [16,17,18] successfully developed and demonstrated the utility of field-based methodology that allowed for the improved inference of environmental persistence of IAVs. Samples held in mesocosms under natural temperature conditions in Alaska, USA, were shown to remain infectious for periods extending up to greater than one year [16], supporting results from previous lab trials and demonstrating the temporal tenacity of IAVs in cold water. Building upon this growing body of the literature, we aimed to extend field-based research to water bodies in Northwestern Minnesota (MN), which serve as important breeding and autumn migratory staging areas for large concentrations of dabbling ducks. Historically, the average prevalence of LP IAVs in ducks at these sites every autumn is 19% (range, 11–33%) [19], and, therefore, there appears to be ample opportunity for the deposition of considerable amounts of viable IAV into the environment, which could remain infectious during the migratory period and over the winter period. Additionally, LP IAVs have previously been isolated from surface waters at these sites [7]. Infectious IAVs may provide a link for the infection of migratory birds using contaminated habitats or serve to re-seed IAV outbreaks when ducks return to these same bodies of water in the spring and summer. The objective of this research was to assess the duration of viral infectivity at seasonal, biologically relevant time points at the local scale for IAV samples held under quasi-natural field conditions at Agassiz National Wildlife Refuge (NWR), Thief Lake Wildlife Management Area (WMA), and Roseau WMA, MN. We performed this research while incorporating additional parallel assessments of viral titers and laboratory-controlled IAV persistence trials so as to refine inference derived from prior work in Alaska [16].

## 2. Materials and Methods

### 2.1. Field Sites, Preparation of Water, Initial Characterization

**Figure 1:**
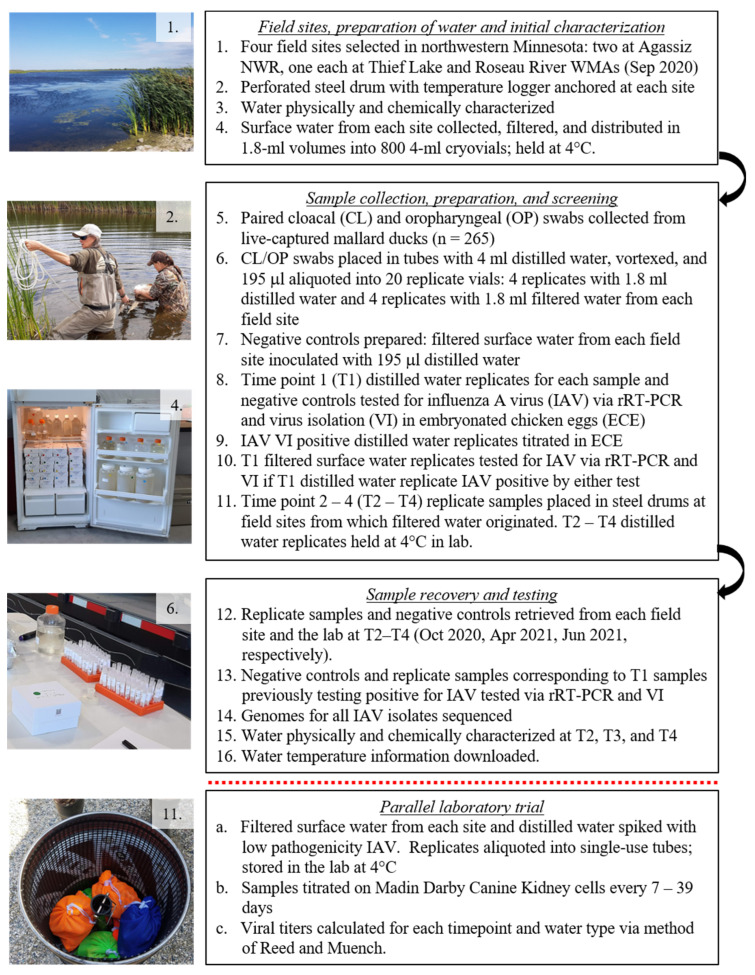
Flow chart (**right**) and photos (**left**) providing a summary of experimental field and laboratory components. Specific details can be found in Section 2.1 (steps 1–4), Section 2.2 (steps 5–7), Section 2.3 (steps 8–10), Section 2.4 (steps 11–16), and Section 2.6 (steps a–c).

Four sites for a field experiment were chosen at locations previously utilized for wild bird IAV surveillance and research at Agassiz NWR (Farmes Pool [48.2703, −96.039], Tamarack Pool [48.3983, −95.9962]), and Thief Lake WMA (48.4556, −95.9535) in Marshall Co., Minnesota (MN) and at Roseau River WMA (48.9557, −96.2097) in Roseau Co., MN, hereafter, water bodies A, B, C, and D, respectively (Figure 1). Briefly, upon site selection, surface water was collected from each location, filtered to 0.22 µm to remove particulates and bacteria, and kept chilled at 4 °C. Filtered water from each site was dispensed into 4 mL cryovials at 1.8 mL volume; distilled water was also aliquoted into 4 mL cryovials at 1.8 mL volume at the laboratory at the University of Georgia (UGA) prior to fieldwork, kept cold, and transported to the MN field lab where it was then used for aliquots of duck swab materials, as described in Section 2.2. 

A multiparameter water quality sonde (Yellow Springs Instruments [YSI], Yellow Springs, Ohio) was used in situ to measure field parameters, including pH, temperature, specific conductance, and dissolved oxygen (DO). In addition, approximately 700 mL of both unfiltered and 0.45 µm filtered water was also collected from each water body in the field at the initiation of this trial [26 August 2020] and sent to the United States Geological Survey (USGS) Redox Chemistry Laboratory (Boulder, CO, USA) for further chemical characterization, as previously described [17].

### 2.2. Sample Collection

Paired cloacal and oropharyngeal (CL/OP) swabs were collected from 266 live captured mallards (*Anas platyrhynchos*) from 25 August to 19 September 2020 (UGA Institutional Animal Care and Use Committee approval A2019 04-001-Y2-A2, U.S. Department of the Interior, U.S. Fish and Wildlife Service Scientific Collection Permit MB53692D) and placed in 8 mL cold, distilled water. Swabs in distilled water were kept at 4 °C until processing, at which point they were vortexed, and supernatants were aliquoted at 195 µL volume into four tubes of 1.8 mL of 0.22 µm filtered water from each water body (A, B, C, or D) plus four tubes of distilled water. One aliquot of each CL/OP swab (initial time point [2 September–19 September 2020], hereafter T1) in filtered water from each water body was immediately sent to the processing laboratory at UGA for cold storage until testing. All distilled water samples were also shipped to the processing laboratory for immediate testing (T1) or laboratory storage at 4 °C. All other aliquots of CL/OP swabs in filtered water (hereafter T2, T3, and T4) from all water bodies (A, B, C, or D) were deployed in the environment, as outlined in Methods Section 2.4. Two replicate negative control tubes (filtered natural water only) from each site for quality assurance were also included for T1 sample processing.

### 2.3. Initial Sample Processing

Each T1 distilled water aliquot (*n* = 266) was screened by real-time reverse transcriptase PCR (rrt-PCR) for the conserved matrix gene of IAVs, as previously described [20]. All distilled water samples with a cycle threshold (Ct) value <45 were considered positive for IAV viral RNA; virus isolation (VI) in embryonated chicken eggs (ECE) was also carried out on all T1 distilled water samples. Briefly, prior to VI, each tube of water was dosed with antibiotic/antimycotic solution (penicillin G: 200 units/mL, Streptomycin: 0.2 mg/mL, and Amphotericin B: 0.5 μg/mL; Sigma-Aldrich, St. Louis, MO, USA), vortexed vigorously, and centrifuged at 3000 rpm × 15 min. Approximately 1.0 mL of supernatant was then evenly divided into the amnioallantoic cavity of three ECEs and allowed to incubate at 37 °C for 5 days. Amnioallantoic fluids were tested via hemagglutination assay (HA) with 0.5% chicken red blood cells [21]; nucleic acids were extracted from all HA-positive samples and then screened in rrt-PCR targeting IAV matrix gene, as described [20].

When IAV was detected by rrt-PCR and/or by VI in distilled water samples, all replicate mesocosm samples (A, B, C, and D) were then subjected to VI, as described above, approximately one week later. Additionally, 64 of 65 distilled water samples that were IAV VI-positive at T1 were also subjected to end-point titration in ECE one to two weeks after initial VI, and the median egg infectious dose (EID_50_/mL) was calculated for each, based on Reed and Muench [22]. The minimum detectable limit for this procedure is 10^0.8^ EID_50_/mL.

### 2.4. Sample Deployment and Retrieval in Natural Water Bodies and Lab Testing

A perforated steel drum containing a Hobo^®^ TidbiT^®^ temperature logger (Onset Computer Corp.; Bourne, MA, USA) was deployed into each water body at a depth of approximately 1 m, as has been previously described [16,17]. Replicate sample tubes for T2, T3, and T4 were contained within mesh bags inside each drum in each water body and deployed between 3 September and 19 September 2020; all tubes were held at 4 °C from the point of collection, with aliquoting of swab materials and until deployment into the environment. Quality assurance/control samples included one tube of uninoculated filtered water for each water body (A–D) for retrieval at T2 and T3. Replicate samples were retrieved at three ecologically relevant time points: ice formation T2 (26 October 2020, 38–62 days post initial sample collection [dpi]); ice out T3 (8 April 2021; 202–226 dpi); and summer T4 (24 June 2021; 279–303 dpi). After the collection of samples for each time point T2 and T3, care was taken to confirm that the drum was still properly anchored at the appropriate depth (approximately 1 m) in the water body. The steel drum and all contents, including the HOBO data logger, were removed from each water body at the conclusion of this study (T4). 

All T2, T3, and T4 samples were sent on ice packs to the processing lab immediately after retrieval. All distilled water samples were removed from 4 °C in the lab within 0–18 days of field sample retrieval (T2: 0–18 days; T3: 0–6 days; T4: 0–8 days). To account for potential heterogeneity across samples, at T2, all replicates (A–D and distilled water) were subjected to VI in ECE if any replicate (A–D and/or distilled water) was IAV rrt-PCR or hemagglutination assay positive at T1. T3 and T4 samples were treated similarly; if any replicates were IAV rrt-PCR or hemagglutination assay was positive at the previous time point, VI was performed for all replicate treatments for that swab sample. Viral quantification was not carried out on T2, T3, and T4 samples, given laboratory and resource constraints.

### 2.5. Analysis and Interpretation

To confirm the results of VI and to identify any potential artifacts resulting from sampling handling or processing procedures, viral isolates were genomically sequenced, and genetic similarity was compared among corresponding replicate samples per procedures previously reported in the summary of a similar field experiment [16]. Genomes of IAVs obtained from all T1 virus isolation replicates derived from the same initial CL/OP swab were first compared; only samples for which viruses shared >99% identity among all replicates (two or more) were used in the assessment of persistence, as was described previously [16]. Resultant viral genomes from IAV isolates obtained from replicates recovered during subsequent sampling efforts (T2, T3, T4) that shared >99% identity with the corresponding T1 genome sample were inferred to have remained infectious at the time of sample retrieval from the natural water body (or for distilled water samples from the laboratory refrigerator). Stringency criteria pertaining to the interpretation of mixed infections were applied as previously described [16].

Kaplan–Meier survival analysis [23] was used to assess potential differences in the persistence of infectious IAVs among replicates held in each of four mesocosms (A, B, C, or D) and the laboratory (distilled water) over time. The start date, which varied among samples, was considered as T1, and the survival endpoint was the mean date between the last time point during which the IAV was isolated and the time point at which infectious IAV was not isolated per replicate. The survival package [24] in R [25] was used to calculate survival curves and confidence intervals.

### 2.6. Laboratory Persistence Trial

The persistence of a duck-derived, egg-propagated LP H3N8 virus held in filtered waters under cold conditions in the laboratory (approximately 4 °C, refrigerator) was assessed to refine inference on variability in viral persistence among water bodies. Briefly, 500 mL of each 0.22 µm filtered natural water was brought into the laboratory and spiked with approximately 104 median tissue culture infectious dose (TCID_50_)/mL of first ECE passage A/mallard/Minnesota/2018(H3N8); a volume of distilled water was also spiked at the same approximate concentration. Spiked waters (filtered and distilled) were then aliquoted at 1 mL volume into individual-use snap-cap tubes and, to maintain surrounding temperature, placed in a plastic container filled with water in a laboratory refrigerator at approximately 4 °C. Samples were titrated on Madin-Darby Canine Kidney (MDCK, CCL-34, American Type Culture Collection, Manassas, VA, USA) cells, as previously described [14], upon experimental setup and then every 7–39 days until the conclusion of the trial at 241 days; viral titers were calculated according to the method of Reed and Muench [22]. Linear regression was used to determine a 90% reduction time (Rt) for each treatment that demonstrated more than a 1 log10 TCID_50_/mL reduction in viral titer. Rt values correspond to the time required for a 1 log10 TCID_50_/mL decrease in viral titer. The minimum detectable limit for this procedure is 10^1.77^ TCID_50_/mL.

## 3. Results

### 3.1. Physical and Chemical Characterization of Water Bodies

**Figure 2:**
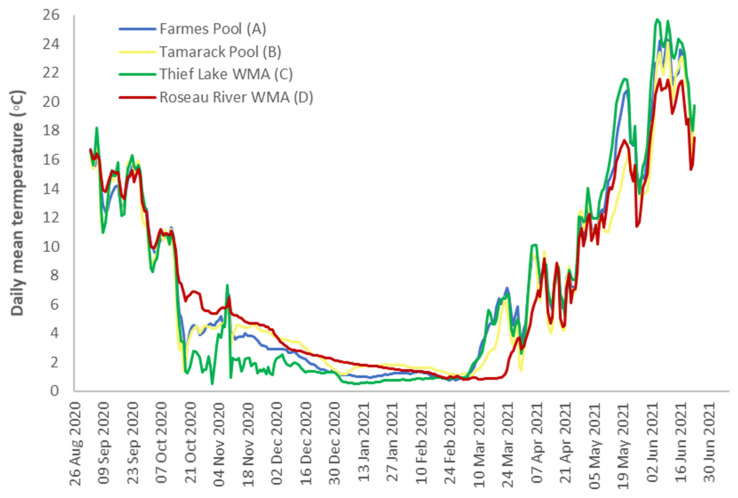
Mean daily surface water temperature for field sites at Agassiz National Wildlife Refuge: Farmes Pool (A, blue line) and Tamarack Pool (B, yellow line); Thief Lake WMA (C, green line); and Roseau River WMA (D, red line) in Northwestern Minnesota, in which samples were held for 279–303 days.

Water temperatures at time point T1 ranged from 13.87 to 16.71 °C and declined relatively quickly to less than 10 °C by 2 October 2020 (16–40 dpi) [26]. Mean daily temperatures were relatively cool and consistent across all water bodies through time point T3 (4.29–4.95 °C) (Figure 2). Temperatures recorded in all water bodies did not drop below 0 °C at any point. At T2, mean temperature readings ranged from 2.35 to 6.72 °C. Mean daily temperatures between T3 and T4 ranged from 13.58 to 15.78 °C, and by 4 June 2021, exceeded 20 °C for all water bodies, with a maximum recording of 25.73 °C at Thief Lake WMA (C) on 5 June 2021. 

**Figure 3:**
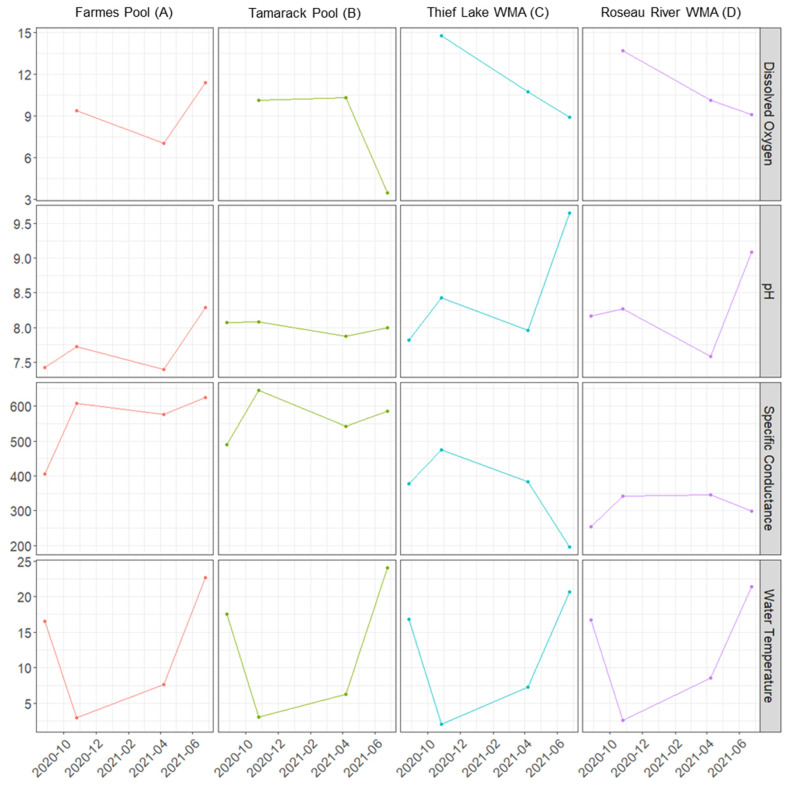
Dissolved oxygen (DO), pH, specific conductance, and water temperatures of surface waters, as measured by Yellow Springs Instruments water quality sonde at each of four time points or at time points 2, 3, and 4 for DO in Minnesota water bodies, in which samples were contained.

Surface water pH measurements at T1 were near neutral for all water bodies, ranging from 7.42 to 8.17, as measured by the YSI probe (Figure 3). Roseau River WMA (D) consistently had the highest pH measurements; all natural water bodies were slightly basic (pH range 8.0–9.65) upon sampling of surface waters at T4. Total dissolved nitrogen (TDN) (range 1.2–60.0 mg/L) and sulfate (range 0.10–160 mg/L) measures were within the expected range for wetlands (Figures S1 and S3); freshwater TDN concentrations typically range from 0.134 to 67.6 mg/L, and sulfate from 0.08 to 8320 mg/L [27]. No consistent trends were apparent for major ions (calcium, magnesium, potassium, phosphorous, silicon dioxide, and sodium) or trace metals (with the exception of elevated arsenic at Roseau River WMA (D)) across and between sites (Figure S2). Alkalinity was reduced at Thief Lake WMA (C) compared to other locations at all time points, and sulfate and chlorine measurements were consistently low at Thief Lake WMA (C) and Roseau River WMA (D) (Figure S3). Specific conductance was elevated at Agassiz NWR water bodies (Farmes Pool, A and Tamarack Pool, B) compared to Thief Lake WMA (C) and Roseau River WMA (D), and dissolved oxygen measures were elevated at all time points at Thief Lake WMA (C) and Roseau River WMA (D) (Figure 3). Chemical and physical properties were not assessed for closed individual replicate samples at any time point.

### 3.2. Initial Sample Analysis

**Figure 4:**
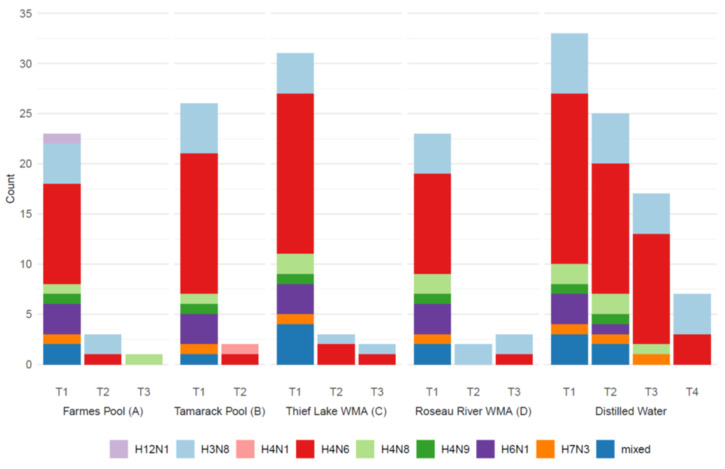
Number of sample replicates confirmed to contain infectious influenza A viruses (IAV) upon retrieval from each natural water treatment (Farmes Pool = A; Tamarack Pool = B; Thief Lake WMA = C; Roseau River WMA = D) at four time points (T1 = 2–19 September 2020; T2 = 26 October 2020; T3 = 8 April 2021; T4 = 24 June 2021) or from distilled water 0–18 days after T2, T3, and T4 retrieval from natural water bodies. Subtype combinations for recovered IAV, determined by genomic characterization, are shown for each location and time point.

From 266 distilled water samples collected at T1, low pathogenicity IAV was identified in 22 (7.9%) by IAV matrix (M) rrt-PCR and isolated from 65 (24.4%) through VI in ECE. Infectious IAV was not isolated from seven of the IAVM rrt-PCR positive samples (7/22; 32%), and viral RNA was not detected in 52 of the VI-positive initial distilled water samples (52/65; 80%). Of the 65 isolates, 36 met the criteria for inclusion in the assessment of the longevity of viral infectivity (28 were only isolated from distilled water, and one was isolated only from a single replicate held in the natural water body and was excluded). Subtypes H4N6 (17/36; 47%) and H3N8 (6/36; 17%) were the most commonly identified. Three H6N1, two H4N8, one of each H4N9 and LP H7N3, and several mixed subtype viruses were also detected. An H12N1 virus was identified in Farmes Pool (A) at T1 and as part of mixed infection in the corresponding distilled water replicate (Figure 4). There was no evidence of viral RNA (by rrt-PCR) nor infectious IAVs (by VI) in replicate tubes of filtered water only (without the addition of swab material) at T1, which served as negative field controls.

### 3.3. Subsequent Testing of Recovered Samples

From replicate samples tested from each mesocosm and distilled water held under laboratory conditions, viral recovery decreased through time upon retrieval at subsequent time points. For example, from an initial 23 infectious IAVs identified at T1 in Farmes Pool (A), viral recovery was reduced to three isolates at T2 and one at T3 (Figure 4). Similar trends of declines in infectious IAVs were found among replicates held in mesocosms in Tamarack Pool (B), Thief Lake WMA (C), and Roseau River WMA (D) (Figure 4). No infectious IAVs were identified in any samples from mesocosms during the final collection effort (T4); in contrast, seven infectious IAVs were isolated from replicate distilled water samples at T4, including four H3N8 and three H4N6 IAVs (Figure 4). There was no evidence of viral RNA (by rrt-PCR) nor infectious IAVs (by VI) in replicate tubes of filtered water only (i.e., without the addition of swab material) retrieved from the environment at T2 and T3, which, again, served as negative field controls.

### 3.4. Initial Distilled Water Sample Titration and Re-Isolation Success

**Figure 5:**
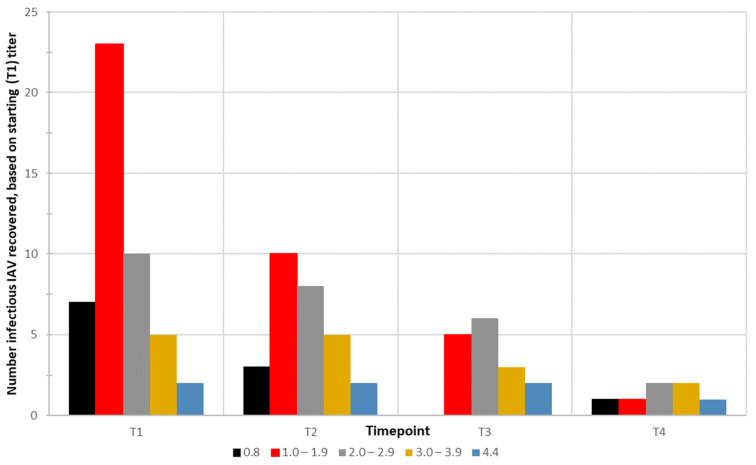
The number of infectious influenza A viruses (IAVs) recovered from mesocosms at each time point (T1–T4), based on initial (T1) starting titer, measured as embryo infectious dose50(EID_50_)/mL. Samples are grouped based on titer at T1 (0.8 EID_50_/mL: black, 1.0–1.9 EID_50_/mL: red, 2.0–2.9 EID_50_/mL: grey, 3.0–3.9 EID_50_/mL: orange, and 4.4 EID_50_/mL: blue). Titer ranges referenced at T2, T3, and T4 are based on the titer determined at T1 and do not refer to viral quantification at those respective later time points.

Of the 65 T1 distilled water samples with infectious IAV, 64 were titrated in ECE, while one sample (sample 197) had inadequate volume for titration. Of these 64, 17 (27%) had viral titers below the limit of detection (10^0.8^ EID_50_/mL). For the remaining infectious T1 samples in distilled water, viral titers ranged from 10^0.8–4.4^ EID_50_/mL. Of the seven samples that had a starting titer of 10^0.8^ EID_50_/mL, three were re-isolated at T2, none were isolated at T3, and one was isolated at T4. Of the 23 samples with initial titers between 10^1.0–1.9^ EID_50_/mL, five and one remained infectious and were detected at T3 and T4, respectively. Six and two of the ten samples with a starting titer of 10^2.0–2.9^ EID_50_/mL were isolated at T3 and T4, respectively. Within the highest titers (10^3.0–4.4^ EID_50_/mL), 100% (7 of 7) of samples were isolated at T2, five of seven at T3, and three at T4 (Figure 5).

Among IAV with the longest viability in the field experiment, the virus within sample 30 (H4N6 subtype IAV), with an initial starting titer of 10^4.4^ EID_50_/mL, could be isolated at T3 from Farmes Pool (A), Thief Lake WMA (C), and Roseau River WMA (D), and remained infectious in distilled water for at least 310 days (through the T4 collection). The IAVs within sample 253 (H3N8; starting titer of 10^3.0^ EID_50_/mL) remained infectious in Farmes Pool (A) through collection at T2, in Roseau River WMA (D) through collection at T3 (for at least 266 days), and was isolated at T3 from Thief Lake WMA (C) despite not being detected at T2 at that same location. The IAV within sample 253 (H3N8) also remained infectious in distilled water until final retrieval at T4. The IAV within sample 148 (H3N8), with a starting titer of 10^2.2^ EID_50_/mL, remained infectious in Roseau River WMA (D) through collection at T3 (for at least 266 days) and in distilled water until retrieval at T4. The IAV within sample 246 (H3N8) with a starting titer of 10^1.3^ EID_50_/mL was identified as infectious from replicates for each water body at T1, from distilled water only at T2, but only from Thief Lake WMA (C) water at T3.

### 3.5. Survival Analysis

**Figure 6:**
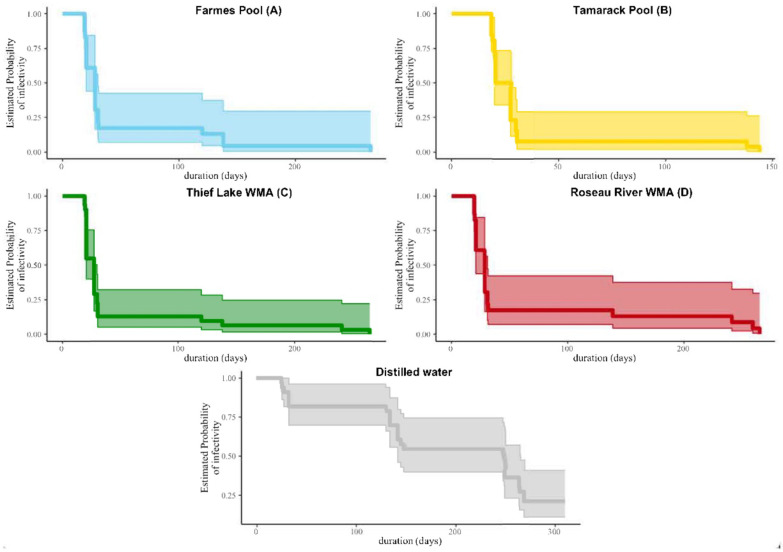
The estimated probability of infectivity of influenza A viruses across time, as depicted by Kaplan–Meier survival curves. Curves for distilled water treatments are based on a temperature of 4C in the laboratory, while those for each of the field sites in Northwestern Minnesota are based on ambient environmental temperatures. The 95% confidence intervals for each curve are shown by the shaded areas.

A pattern of decreasing viral infectivity with time was noted for samples held within mesocosms at all four natural water bodies and distilled water replicates, as assessed by Kaplan–Meier survival analyses [23] (Figure 6). For all-natural water bodies, estimates of infectivity for IAVs decreased below the first probability quartile (0.25) by day 40. In contrast, the probability of infectivity of IAVs maintained in distilled water conditions in the lab remained between the second and third quartiles (0.25–0.75) past day 200.

### 3.6. Laboratory Persistence Trial

Decreasing infectivity of egg-propagated LP H3N8 through time was noted in all natural and distilled water treatments held at 4 °C over the course of the approximately 8-month laboratory trial (Table 1). In the distilled water treatment, the Rt value (time in days required for a one-log decrease in viral titer) was determined to be approximately 139 days, while Rt values for the natural waters were markedly lower and ranged from 47.4 days (Roseau River WMA, D) to 95.2 days (Thief Lake WMA, C) (Table 1).

## 4. Discussion

While a fecal–oral route has long been considered an important mode of IAV transmission in avian species, the role that viral persistence in natural water settings plays in such transmission mechanisms is not well understood. Previous work has shown that some LP IAVs can remain infective for periods of time, exceeding one year in mesocosms in northern wetlands of North America [16], and are capable of infecting mallard ducks when held under quasi-ambient environmental conditions over the wintering period [17]. Here, subtypes H3N8, H4N6, and H4N8 IAV were also found to remain infectious over the wintering period or for at least 202 days (from T1 until T3, from mid-September 2020 to early April 2021) when held under quasi-ambient environmental conditions in Northwestern Minnesota water bodies. While this timeframe is less than the maximum duration of infectivity for some IAVs held under similar quasi-field conditions in Alaska, as reported previously [16], prior studies aiming to assess the infectivity of IAVs in natural water bodies did not quantify or account for viral titer of the initial T1 samples [16,17,18].

The duration of infectivity was the greatest in those samples with the highest initial viral titer. The 4 °C laboratory trial carried out as part of this work indicated that Rt (time for one log reduction in viral titer) values, based on titration on MDCK cells, ranged from 47–95 days for filtered natural waters. Though we cannot directly compare TCID50 and EID50 values, given that many distilled water samples had initial titers near the limit of detection in the ECE model, the marked reduction in IAV recovered from T1 to T2 (38–62 days) and then at T3 (more than 200 days after project initiation) is logical. The highest initial titer in this work was 10^4.4^ EID_50_/mL. Even accounting for the dilution effects of swab materials being submerged in 8 mL water, this starting titer is still lower than has been reported experimentally in Muscovy and mallard duck feces [1,28]. Viruses shed at higher titers may, in fact, have a higher propensity for extended periods of infectivity in natural systems, and as such, it is important to account for viral quantities when considering persistence relative to environmental testing.

The marked decrease in viral recovery from T1 to T2 (between 38 and 62 days) for all samples held in mesocosms in this study was more pronounced than was described for similar work carried out in Alaska water bodies [16]. It should be noted, however, that water temperatures were, on average, 4.8–7 °C colder at the initiation of the Alaska trial, and the higher T1 temperatures reported in this work may have resulted in quicker viral decay and decreased recovery from T1 to T2. Here, the duration of infectivity of IAV retained in distilled water treatments at a consistently cold temperature in the lab exceeded that of viruses held in mesocosms (filtered natural waters under environmentally relevant temperatures). Seven of 30 (23%) viruses detected at T1 (three H4N6 and four H3N8) remained infectious under these laboratory-controlled conditions for at least 279 days (six months). While natural waters remained relatively cool over the course of this study, based on laboratory models, viral persistence is known to decrease with increasing temperatures and freeze–thaw events [7,13,14,29]. Even at temperatures lower than 17 °C, individual viruses show noticeable differences in persistence in laboratory models [6,14]. While temperatures were relatively cold in all-natural water bodies reported here, through T2, temperature fluctuations throughout the autumn until ice formed at the surface of the water in the winter could have negatively impacted infectivity. Further, the water chemistry of the filtered natural waters held in individual closed tubes likely had an effect on viral tenacity over time. Previous work assessing IAV persistence in filtered natural surface waters in a laboratory demonstrated decreasing viral persistence with increasing sulfate concentrations [30]. While five infectious IAVs were recovered from Thief and Roseau mesocosms at T3, mesocosms at Agassiz NWR, with higher overall sulfate levels only yielded one IAV at this same time point. Strain variation also has been shown to play a role in viral stability [31]. It should be noted that nearly all viruses (29/30; 97%) that persisted until sampling at T3 in either distilled water or mesocosms were of the H3 or H4 hemagglutinin and N6 or N8 neuraminidase subtypes. These are the most commonly identified subtypes in North American ducks in the autumn [19,32,33]. The relationship between the tenacity of individual subtype combinations and seasonal patterns of IAV prevalence and diversity in wild birds remains unexplored. 

The environmental persistence of avian IAVs likely plays a critical role in their transmission potential, especially when this ability to remain infectious can span seasons. Recent work suggests that infection via the fecal–oral route via surface water in mallards may require a relatively low amount of IAV to cause infection (10^0.1^ TCID_50_/mL) [34]. IAVs that remain infectious for extended periods of time, even at low titers in the environment, may contribute to infection or even outbreaks when susceptible birds return to important mid- to high-latitude breeding and staging sites such as those studied in this work. Unraveling the myriad of components that drive such mechanisms in natural settings is complicated; the work presented here helps to delineate factors and refine methodologies that can help better understand IAV in the environment. The discordance between rrt-PCR and VI results reported here is not uncommon for environmental work [16,17,18,35] and may be due, in part, to the presence of the PCR inhibitors in water from mesocosms interfering with amplification and/or detection of viral RNAs. Additionally, the isolation of IAVs from samples at the later but not preceding time points (e.g., 253 at T3 from Thief Lake WMA), while illustrating another complication of this work, is not all that surprising given that each closed tube serves as its own unique microenvironment, potentially with different initial distribution in the number of infectious IAVs within it. Results such as these add more layers of complexity to the resultant interpretation and inference related to infectivity. Research efforts have attempted to refine methods for the identification and/or isolation of infectious IAVs or viral RNAs from the environment [8,35,36,37], but extrapolation of such results is limited by the complex biotic and abiotic factors that drive viral persistence. The circulation of highly pathogenic Gs/GD lineage H5 IAV for nearly two years in North America highlights the need to better define and scrutinize methods in both laboratory and natural settings, which aim to define environmental components important in IAV maintenance and transmission. Combining such environmental approaches with active wild bird research-based surveillance has utility when responding to outbreaks of IAVs among species of conservation concern. For example, incorporating information on the infectivity of viruses detected among habitats used by wildlife of conservation concern may help to inform whether and how managers choose to monitor species and sites and perceive the risk of continued or future outbreak events.

## 5. Conclusions

In summary, the results of this investigation refine inference on the persistence of infectious IAVs in natural wetlands. The findings of this investigation support the premise that the duration of IAV infectivity among contaminated waters is a function of the amount of virus deposited, strain-specific viral characteristics, ambient temperature, and water chemistry. Though environmental persistence likely plays an important role in the transmission of the virus among wild bird hosts via a fecal–oral route, complex interactions between viruses and the environment complicate our ability to quantify the relative contributions of this mechanism to the maintenance of viruses among wild bird reservoirs. Future research might be most impactful by developing model systems using Gs/GD lineage H5 IAV maintained within waters with chemical and physical properties representative of habitats used by large concentrations of aquatic birds recognized as reservoirs of IAVs. Such models may facilitate better ascertainment of environmental exposure risk to infectious Gs/GD lineage H5 IAVs during the ongoing panzootic.

## Figures and Tables

**Table 1:** Viral titers for each filtered natural and distilled water treatment inoculated with a low pathogenicity H3N8 virus and held at 4 °C as part of the experimental laboratory persistence trial. The limit of detection is 10^1.77^ TCID_50_/mL.

	Titer (TCID_50_/mL) ^#^													
	Days Post Inoculation Water Samples													
Water Treatment	0	26	42	50	78	91	118	146	167	174	202	241	Regression Equation [Rt ^##^ Value (Days)]	R^2^
Farmes Pool (A)	4.23	3.63	3.28	2.56	1.93	1.93	1.86	1.77	<1.77	<1.77	<1.77	<1.77	y = −0.018x + 3.87 [56.18]	0.834
Tamarac Pool (B)	4.37	3.90	3.01	2.56	2.02	2.52	2.05	1.77	1.80	<1.77	<1.77	<1.77	y = −0.015x + 3.84 [67.57]	0.790
Thief Lake WMA (C)	4.33	4.17	3.81	3.32	3.28	3.52	2.81	2.52	2.59	1.90	2.52	1.86	y = −0.010x + 4.19 [95.24]	0.892
Roseau River WMA (D)	4.37	3.81	3.57	3.28	2.76	2.47	1.87	<1.77	<1.77	<1.77	<1.77	<1.77	y = −0.021x + 4.38 [47.39]	0.998
Distilled water	3.62	3.23	2.68	2.47	2.63	2.73	2.47	2.05	2.00	1.84	1.79	1.77	y = −0.007x + 3.24 [138.89]	0.854

^#^ Titer, as reported in tissue culture infectious dose_50_/milliliter (TCID_50_/mL). ^##^ Reduction time (Rt): time required for a decrease in viral titer by 1 log_10_ TCID_50_/mL.

## Data Availability

The data presented in this study are available as a USGS data release [26] [Scott et al. (2024)]. Virus genomes are available on GenBank under accession numbers OQ095578-OQ095913 and OR995000-OR995183.

## Supplementary Materials

The following supporting information can be downloaded at https://www.mdpi.com/article/10.3390/pathogens13050406/s1, Figure S1: Plots of nitrogen levels (ammonium, nitrate, nitrate + nitrite, nitrate and total dissolved nitrogen for surface waters of four sampling sites (Farmes Pool (A), Tamarack Pool (B), Thief Lake WMA (C), and Roseau River WMA (D)) and at four time points (T1 = September 2020; T2 = October 2020; T3 = April 2021; T4 = June 2021) [26]; Figure S2: Plots of dissolved and total major ions and trace metals, as defined in legend and measured in mg/L for surface waters of four sampling sites (Farmes Pool (A), Tamarack Pool (B), Thief Lake WMA (C), and Roseau River WMA (D)) and at four time points (T1 = September 2020; T2 = October 2020; T3 = April 2021; T4 = June 2021). Note that all non-detections have been removed, but these data are included in the data release [26]; Figure S3: Plots of alkalinity, chloride, DOC, fluorine, and sulfate, as measured in mg/L for surface waters of four sampling sites (Farmes Pool (A), Tamarack Pool (B), Thief Lake WMA (C), and Roseau River WMA (D)) and at four time points (T1 = September 2020; T2 = October 2020; T3 = April 2021, T4 = June 2021) [26].
