# Supplementary material for: Infectivity of Wild-Bird Origin Influenza A Viruses in Minnesota Wetlands across Seasons
Source: Pathogens. 2024 May 14;13(5):406. doi: 10.3390/pathogens13050406 (PMC11124429; doi:10.3390/pathogens13050406)
Supplement: Supplementary file 1 [file pathogens-13-00406-s001.zip › Figure S1_Nitrogen.pdf]

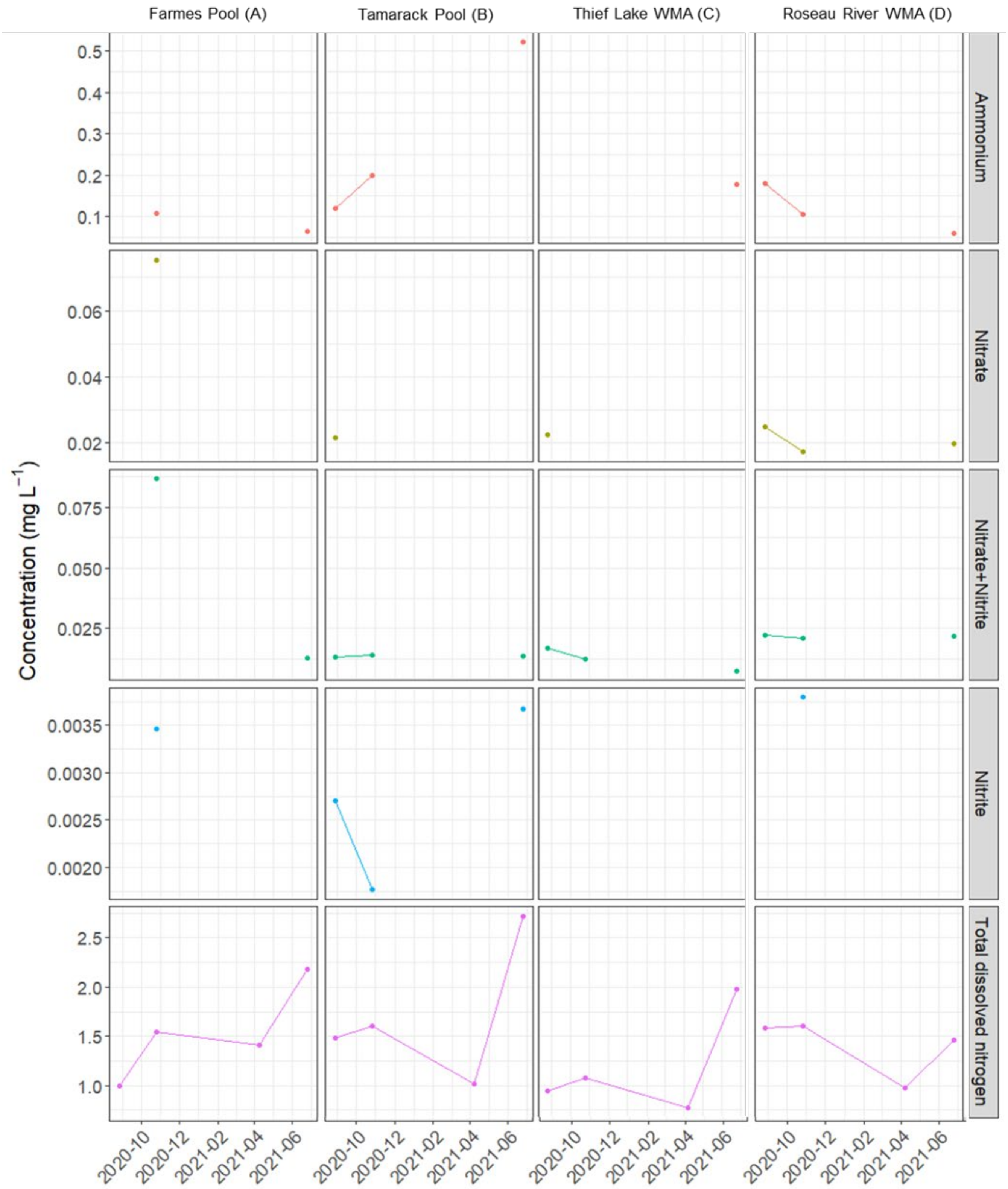

Supplemental Figure S1. Plots of nitrogen levels (ammonium, nitrate, nitrate + nitrite, nitrate and total dissolved nitrogen for surface waters of four sampling sites (Farmes Pool (A), Tamarack Pool (B), Thief Lake WMA (C) and Roseau River WMA (D)), and at four time points (T1 = September 2020; T2 = October 2020; T3 = April 2021, T4 = June 2021).
