# Supplementary material for: Infectivity of Wild-Bird Origin Influenza A Viruses in Minnesota Wetlands across Seasons
Source: Pathogens. 2024 May 14;13(5):406. doi: 10.3390/pathogens13050406 (PMC11124429; doi:10.3390/pathogens13050406)
Supplement: Supplementary file 1 [file pathogens-13-00406-s001.zip › Figure S2-ions.pdf]

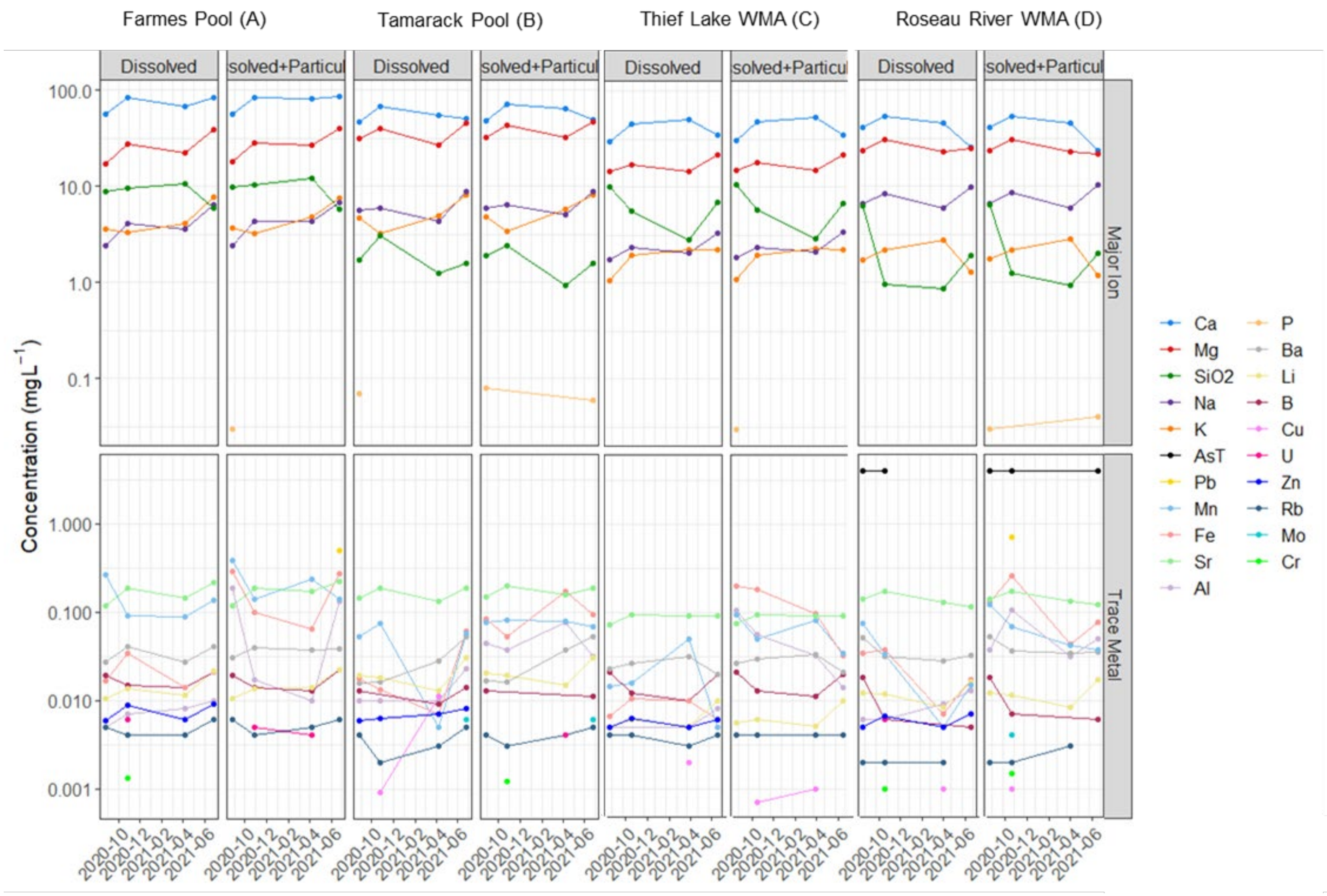

Supplemental Figure S2. Plots of dissolved and total major ions and trace metals, as defined in legend and measured in mg/L for surface waters of four sampling sites (Farmes Pool (A), Tamarack Pool (B), Thief Lake WMA (C) and Roseau River WMA (D)), and at four time points (T1 = September 2020; T2 = October 2020; T3 = April 2021, T4 = June 2021). Note that all non-detections have been removed, but these data are including in the data release at [REFERENCE].
